# A network meta-analysis of first-line treatment options for patients with Child-Pugh class B functional hepatocellular carcinoma: comparison of efficacy and safety

**DOI:** 10.3389/fphar.2025.1705502

**Published:** 2026-01-02

**Authors:** Yu-Xuan Zhang, Jia-Yi Wu, Jun-Yi Wu, Zhen-Xin Zeng, Rong-Jian Pan, Xiang-Ye Ou, Yi-Nan Li, Mao-Lin Yan

**Affiliations:** Shengli Clinical Medical College of Fujian Medical University, Department of Hepatobiliary Pancreatic Surgery, Fujian Provincial Hospital, Fuzhou University Affiliated Provincial Hospital, Fuzhou, Fujian, China

**Keywords:** hepatocellular carcinoma, Child-Pugh class B, network meta-analysis, first-linetreatment, efficacy, safety

## Abstract

**Introduction:**

Management of patients with advanced hepatocellular carcinoma (HCC) and Child-Pugh class B liver function is challenging owing to compromised hepatic functional reserve, affecting treatment selection and outcomes, as many systemic therapies demonstrate altered pharmacokinetics and increased toxicity in this population. Current treatment guidelines predominantly focus on patients with preserved liver function (Child-Pugh A), creating a critical evidence gap for the optimal management of patients with Child-Pugh B liver function. This network meta-analysis (NMA) evaluated first-line therapies for patients with advanced HCC and Child-Pugh B cirrhosis, addressing current evidence gaps to guide optimal treatment selection for this high-risk population.

**Methods:**

The PubMed, Embase, and Cochrane Library databases were searched until 31 October 2024. Randomized controlled trials and prospective cohort studies were included if they involved patients with advanced HCC and Child-Pugh class B liver function, evaluated first-line therapeutic agents, and reported overall survival (OS) and/or progression-free survival (PFS) with 95% confidence intervals. Bayesian NMA was employed to evaluate treatment efficacy and safety.

**Results:**

Eleven studies comprising 2,536 patients were included in the NMA. Lenvatinib exhibited the greatest probability of ideal efficacy for OS, while atezolizumab and bevacizumab combination therapy demonstrated the highest likelihood of optimal performance in terms of PFS. The overall incidence of adverse events (AEs) was 68.58%. The predominant grade 3–4 AEs included hypertension, proteinuria, hand-foot syndrome, and abnormal liver function.

**Conclusion:**

Atezolizumab and bevacizumab combination therapy demonstrated the optimal net benefit regarding PFS and reduced toxicity, whereas lenvatinib monotherapy exhibited the greatest net benefit in OS but with increased toxicity.

## 1 Introduction

Hepatocellular carcinoma (HCC) is a predominant cause of cancer-related mortality worldwide, with persistently elevated incidence and mortality rates. The World Health Organization reports HCC as the sixth most prevalent cancer and the fourth primary cause of cancer-related mortality worldwide (Sung et al., 2021). The onset of HCC is intricately linked to chronic liver ailments, particularly infections by hepatitis B and C viruses, alcoholic liver disease, and non-alcoholic fatty liver disease (Llovet et al., 2021). Owing to the absence of early symptoms, the majority of patients with HCC are diagnosed at advanced stages, resulting in the forfeiture of surgical resection opportunities (Forner et al., 2018).

For patients with advanced HCC, systemic therapy constitutes the primary treatment modality. Liver function status is a critical determinant of treatment options and prognosis. The Child-Pugh classification system is a vital instrument for evaluating liver function, with Child-Pugh class B patients representing a substantial percentage of patients with HCC (Johnson et al., 2015). These patients possess constrained therapeutic alternatives owing to their compromised liver function and reduced treatment tolerance. Consequently, the choice of suitable treatment modalities is essential for enhancing the prognosis of patients with HCC and Child-Pugh class B liver function.

In recent years, the rapid advancement of molecularly targeted therapies and immune checkpoint inhibitors has markedly transformed the treatment landscape of HCC. Sorafenib, the first approved targeted medication for advanced HCC, heralded a new epoch in targeted therapy for HCC (Llovet et al., 2008). Subsequently, lenvatinib demonstrated comparable efficacy to sorafenib in non-inferiority trials, thereby expanding the therapeutic options for HCC (Kudo et al., 2018). In recent years, immune checkpoint inhibitors such as nivolumab and pembrolizumab have demonstrated potential in the treatment of HCC (El-Khoueiry et al., 2017; Zhu et al., 2018). Atezolizumab and bevacizumab combination therapy, demonstrating greater performance than sorafenib in the IMbrave150 study, has established itself as the new standard for first-line treatment of advanced HCC (Finn et al., 2020).

Despite these advancements, the ideal first-line treatment for patients with HCC and Child-Pugh class B liver function remains contentious. The safety and efficacy of immunotherapy in patients with Child-Pugh class B require additional investigation. A small study indicated that patients with Child-Pugh grade B undergoing immunotherapy exhibited an elevated risk of deteriorating liver function (Yau et al., 2022). The efficacy and safety of conventional systemic chemotherapy, molecularly targeted therapies, and emerging immunotherapies have not been sufficiently compared in this population. Consequently, this study sought to systematically evaluate the efficacy (e.g., overall survival [OS] and progression-free survival [PFS]) and safety (e.g., incidence of AEs) of first-line treatment regimens in patients with Child-Pugh class B HCC through a network meta-analysis (NMA), aiming to provide a scientific foundation for clinical practice.

## 2 Methodology

The NMA was conducted in accordance with the Preferred Reporting Items for Systematic Evaluation and Meta-Analyses (PRISMA) guidelines (Hutton et al., 2015).

### 2.1 Data sources and search strategies

A systematic literature search of the PubMed, Embase, and Cochrane Library databases was performed for full-text articles published up to November 2024. Randomized controlled trials (RCTs) evaluating first-line regimens for advanced Child-Pugh class B HCC were identified from the inception of each database to November 2024. The search strategy was (“Sorafenib” OR “Lenvatinib” OR “Cabozantinib” OR “Regorafenib” OR “Tyrosine Kinase Inhibitor” OR “Bevacizumab” OR “Atezolizumab” OR “Pembrolizumab” OR “Durvalumab” OR “Avelumab” OR “Trametinib” OR “Tislelizumab” OR “Ipilimumab” OR “PD-1 inhibitor” OR “PD-L1 inhibitor” OR “CTLA-4 inhibitor” OR “Immune checkpoint inhibitor”) AND (“Hepatocellular Carcinoma” OR “HCC”) AND “Child-Pugh B.” Two evaluators independently conducted the literature searches.

### 2.2 Study selection and data extraction

RCTs were included based on the following criteria using the PICOS system: (i) Participants: patients with advanced HCC exhibiting histopathological, cytological, or clinically diagnosed liver function classified as Child-Pugh class B (adapted from the Chinese guidelines for the diagnosis and treatment of primary HCC) (Department of Medical Administration and National Health and Health Commission of the People’s Republic of China, 2020); (ii) Intervention: systemic regimen as first-line treatment; (iii) Comparison: control group receiving active treatment; and (iv) Outcome: primary outcomes included OS and PFS, while the secondary outcome encompassed treatment-related AEs of any grade.

### 2.3 Quality assessment

The Cochrane Collaboration’s Trial Risk of Bias Assessment Tool (Higgins et al., 2011) was employed to evaluate bias, encompassing randomized sequence generation, allocation concealment, participant and personnel blinding, outcome assessment blinding, incomplete outcome data, selective reporting, and additional biases (Supplementary Figure 1). The studies were independently evaluated by two reviewers, and discrepancies were addressed by a third party.

### 2.4 Statistical analyses

The NMA was conducted using a Bayesian methodology, comparing all treatment regimens to atezolizumab plus bevacizumab combination therapy. This meta-analysis evaluated OS, PFS, and treatment-related AEs in patients with Child-Pugh stage B advanced HCC undergoing various treatment regimens, including comparisons with patients having Child-Pugh class A advanced HCC. For OS and PFS, meta-analyses were conducted using the median survival times with a 95% confidence interval (CI) and permuted variance. We assessed the comparative ranking of therapies for OS and PFS by utilizing their surface under the cumulative ranking curve (SUCRA), which ranged from 0 (indicating that a treatment regimen is unequivocally the least effective) to 1 (indicating that a treatment regimen is unequivocally the most effective). Additionally, we constructed forest plots illustrating the effect sizes and 95% CIs for comparisons of various treatment regimens. League tables displaying pairwise comparisons of OS and PFS for different treatment regimens were also produced. All statistical analyses were conducted using Stata 16.0, with p < 0.05 being statistically significant.

## 3 Results

### 3.1 Literature search and research characteristics

**FIGURE 1:**
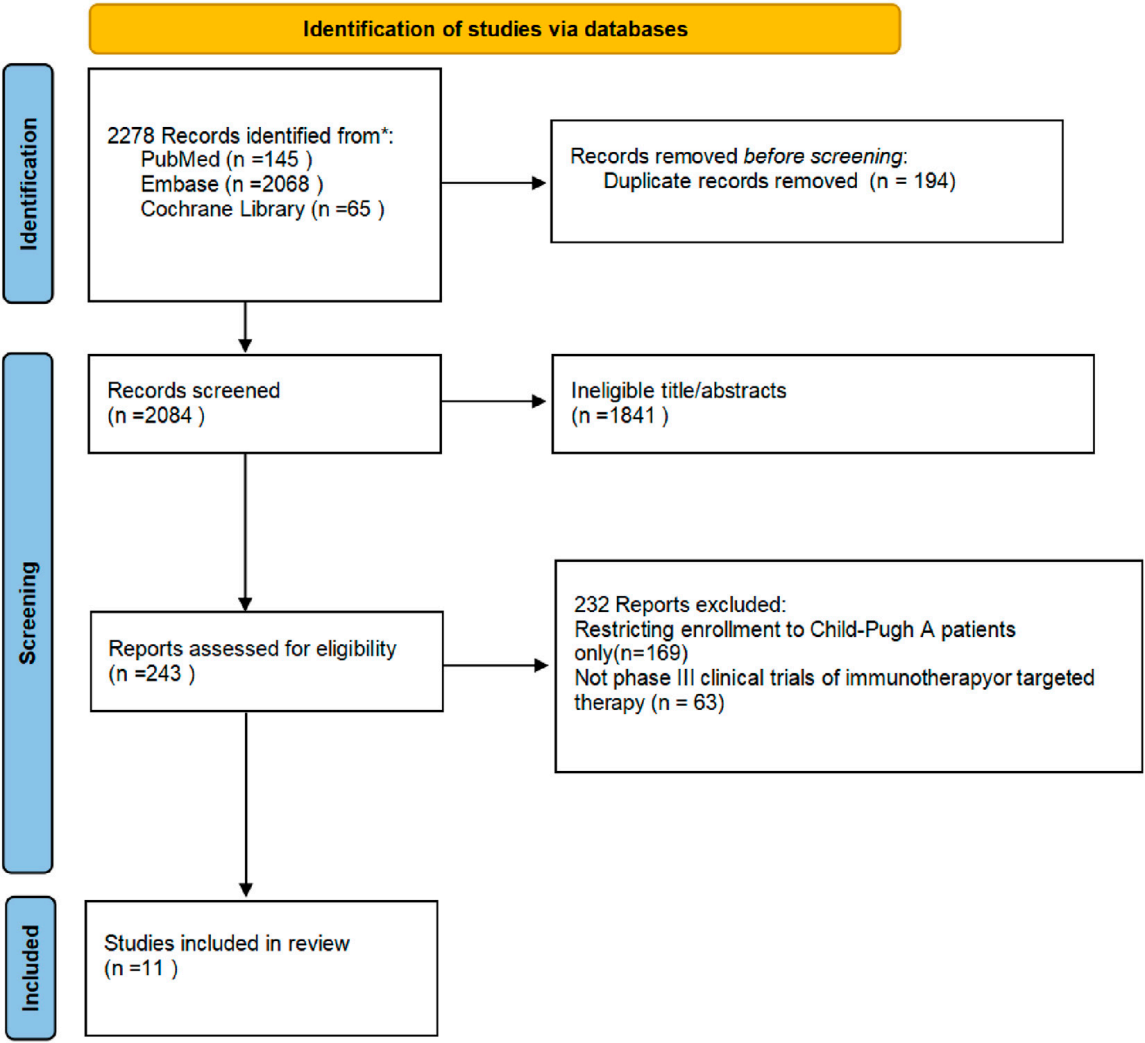
Flowchart of study selection. (PRISMA flow diagram).

**FIGURE 2:**
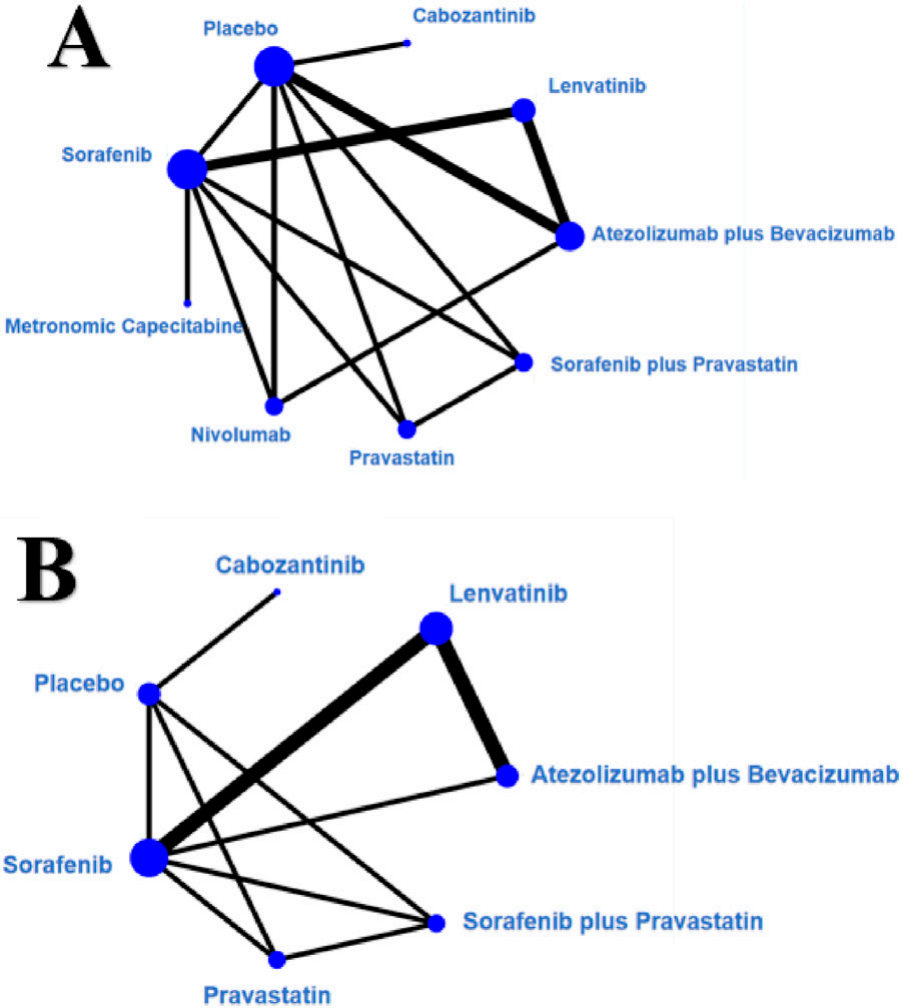
Network diagrams of comparisons of different treatments outcomes in patients with HCC. **(A)** Overall survival. **(B)** Progression-free survival.

The study selection flowchart (PRISMA flow diagram) is shown in Figure 1. Our search identified 18,189 publications, of which 1,219 were initially evaluated and 137 underwent full-text evaluation, culminating in the inclusion of 11 articles (Stefanini et al., 2024; Fulgenzi et al., 2024; Blanc et al., 2021; Park et al., 2022; Chapin et al., 2023; Huynh et al., 2022; El-Khoueiry et al., 2022; Rimini et al., 2023; Ohama et al., 2023; Kikugawa et al., 2024; Fulgenzi et al., 2023). A total of 2,536 patients with advanced HCC classified as Child-Pugh class B were analyzed, encompassing a diverse array of first-line therapeutic agents, including but not limited to sorafenib, lenvatinib, and atezolizumab plus bevacizumab combination therapy. Table 1 illustrates the fundamental characteristics of the included studies, such as sample size, patient age, sex ratio, and other relevant factors. Six studies assessed sorafenib, five examined lenvatinib, two investigated nivolumab, and one analyzed cabozantinib, metronomic capecitabine, and a combination of sorafenib and pravastatin. The median age of the patients ranged from 62 to 73 years. A total of 10 articles presented the median OS with 95% CI over nine dosage regimens: atezolizumab plus bevacizumab, lenvatinib, cabozantinib, placebo, sorafenib, metronomic capecitabine, nivolumab, pravastatin, and sorafenib plus pravastatin. Seven of them presented the median PFS with 95% CI across seven dosage regimens: atezolizumab plus bevacizumab, lenvatinib, cabozantinib, placebo, sorafenib, pravastatin, and sorafenib plus pravastatin. The outcomes of the NMA indicated differences in first-line efficacy across the treatment regimens. Supplementary Figure 3 shows forest plots of OS and PFS, depicting the outcomes of direct treatment comparisons. Furthermore, we developed an evidence network graph (Figure 2) that illustrates the comparative linkages and evidential strengths among treatment alternatives. Nodes signify various treatment measures, while the connecting lines denote comparisons between different treatment options. The thickness of these lines indicates the number of studies that directly compare them.

**TABLE 1:** Patient characteristics.

Study	Arm	Patients, n	Malen (%)	Age, median (IQR)	HBV, n (%)	HCV, n (%)	AFP	BCLC stage, n (%) A B C D			
Anthony B el-khoueiry	Cabozantinib	51	45 (88)	63.0 (22nib)	18 (35)	16 (31)	NR	NR	NR	NR	NR
	Placebo	22	20 (91)	64.5 (505 (5	6 (27)	4 (18)	NR	NR	NR	NR	NR
Hideko ohama	A + T	29	23 (79	72 (67–81)	NR		67.2 (7.0ib)acteris	9 (31)	17 (59)	3 (10)	0
	Lenvatinib	99	74 (75)	64.5 (50ibb)	NR		120.3 (11.1)acterist	33 (33)	61 (62)	5 (5)	0
Benedetta stefanini	Sorafenib	410	338 (82.4)	68.5 (59.21)acte	NR		NR	NR	NR	NR	NR
	Metronomic capecitabine	62	47 (75.8)	68.6 (63.3e)acte	NR		NR	NR	NR	NR	NR
Margherita rimini	Lenvatinib	65	58 (89)	71 (40–91)	15 (23)		NR	0	19 (29)	46 (71)	0
	A + T	152	115 (76)	70 (45–90)	29 (19)		NR	0	55 (36)	97 (64)	0
William J chapin	Nivolumab	79	424 (98)	66.6 (63.1–71.4)	7 (2)	131 (30)	70.1 (9.1–1,091.0)	NR	NR	NR	NR
	Sorafenib	431	78 (99)	70 (45–90)	2 (3)	24 (30)	171.6 (10.8–2,980.3)	NR	NR	NR	NR
C. Fulgenzi	A + T	136	NR	NR	NR		NR	NR	NR	NR	NR
	Placebo	159	NR	NR	NR		NR	NR	NR	NR	NR
Jasmine Huynh	Lenvatinib	60	45 (75.0)	64.0 (26)byn	29 (48.3)	15 (25.0)	NR	NR	NR	NR	NR
	Sorafenib	47	38 (80.9)	64.0 (26) yn	20 (42.6)	12 (25.5)	NR	NR	NR	NR	NR
Min kyung park	Lenvatinib	34	29 (85.3)	62 (55.3)b	26 (76.5)	NR	2,614 (415) ynhct	0 (0)	1 (2.9)	29 (85.3)	4 (11.8)
	Sorafenib	60	52 (86.7)	65 (56(56.	43 (71.7)	NR	2,517 (121(121) yn	0 (0)	4 (6.7)	52 (86.7)	4 (6.7)
Jean-frédéric blanc	Sorafenib	40	37 (90)	67 (51)ib	2 (5)	1 (2)	95 (611,038)	0 (0)	4 (10)	36 (88)	1 (2)
	Pravastatin	38	34 (87)	63 (43)tin	2 (5)	4 (10)	1,462 (48tinynhct	0 (0)	6 (15)	33 (85)	0 (0)
	sorafenib + pravastatin	37	35 (88)	66 (44–82)	2 (5)	2 (5)	38 (84–82)	0 (0)	5 (13)	35 (88)	0 (0)
	Placebo	39	35 (95)	65 (47)82)	1 (3)	0 (0)	50 (14)7,791)	0 (0)	4 (11)	31 (84)	2 (5)
Kikugawa C	Sorafenib	100	82 (82)	70 (61.3–76)	NR		268.5 (14.7–8,597.1)	3 (3)	22 (22)	75 (75)	0 (0)
	Lenvatinib	19	18 (94.7)	64 (58–79)	NR		237.7 (5.3–40990)	0	7 (37)	12 (63)	0 (0)
	A + T	22	19 (86.4)	69.5 (63.8–78)	NR		12.7 (3.6–99.8)	0	9 (41)	13 (59)	0 (0)
Claudia angela maria fulgenzi	Active treatment	187	154 (82)	66 (61–72)	50 (27)	48 (26)	NR	0	33 (18)	154 (82)	0 (0)
	Placebo	158	117 (74)	73 (66–81)	27 (17)	79 (50)	NR	0	13 (8)	145 (92)	0 (0)

AFP, alpha-fetoprotein; BCLC, HBV, hepatitis B virus; HCV, hepatitis C virus; NR, not reported; A + T,Atezolizumab + Bevacizumab; BCLC, barcelona clinic liver cancer stage.

### 3.2 Survival time assessment

**FIGURE 3:**
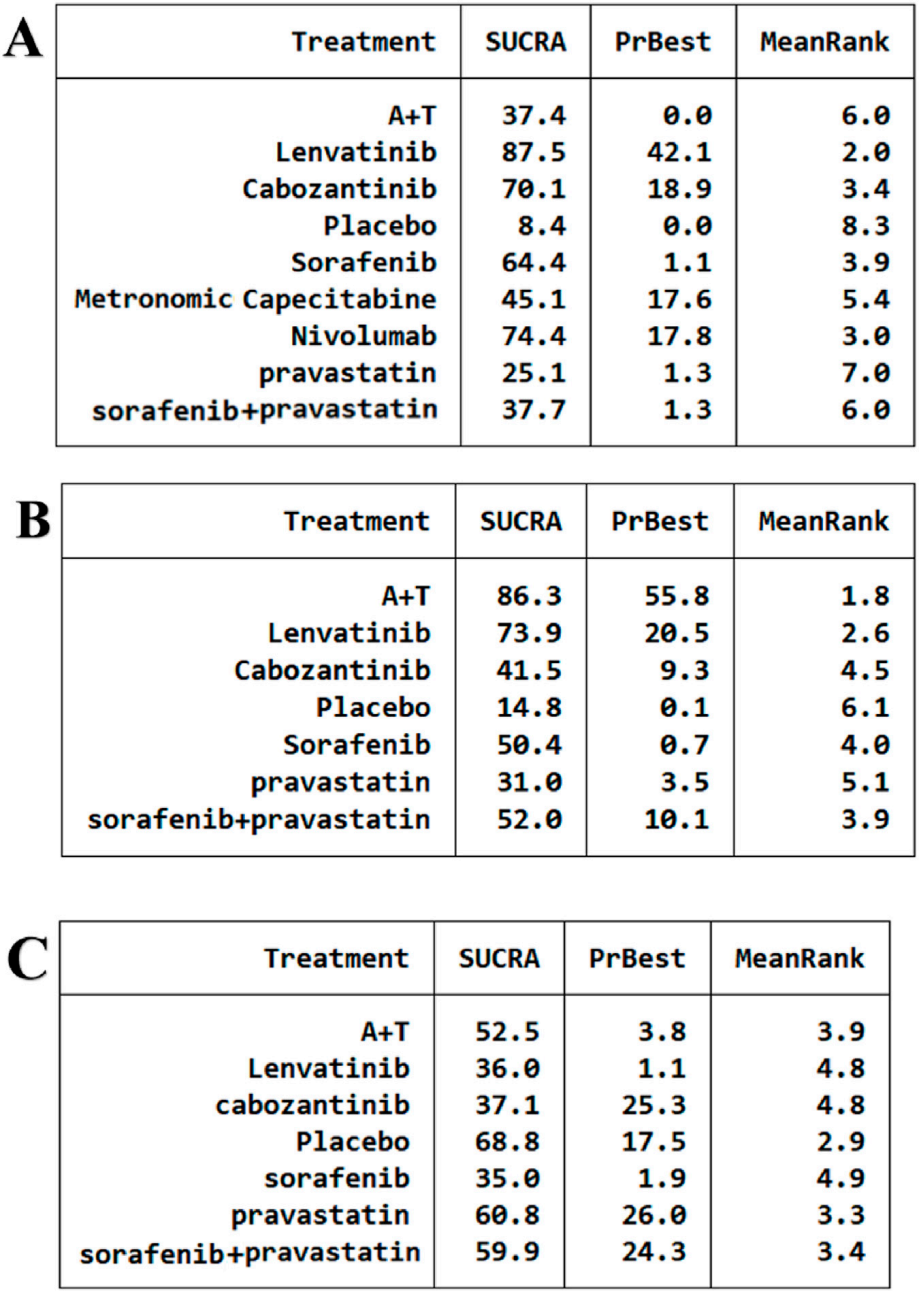
SUCRA values for different regimens. **(A)** Overall survival. **(B)** Progression-free survival. **(C)** Adverse events (AEs).

The forest plot in Supplementary Figure 3A illustrates that the OS advantage of the lenvatinib group was markedly superior to that of the alternative regimens. The median OS logarithmic value for the atezolizumab plus bevacizumab combination therapy was 3.4 (95% CI: 0.43–6.3), indicating a statistically significant survival benefit. SUCRA (Figure 3A) sorting revealed that atezolizumab plus bevacizumab combination therapy had a 42.6% probability of optimal efficacy, suggesting that it was the most favorable treatment option. Nivolumab demonstrated an OS improvement of 1.9 (95% CI: −3.9–7.7), yielding an SUCRA value of 17.6, but the difference was not statistically significant. Except for lenvatinib, none of the other treatment regimens exhibited statistically significant differences compared with atezolizumab plus bevacizumab combination therapy. The hierarchy of efficacy, from highest to lowest, was as follows: lenvatinib, nivolumab, cabozantinib, sorafenib, atezolizumab plus bevacizumab, sorafenib plus pravastatin, pravastatin, metronomic capecitabine, and placebo.

Regarding PFS, the forest plot of Supplementary Figure 3B demonstrates that the atezolizumab plus bevacizumab combination therapy exhibited a notable advantage, while lenvatinib showed a median PFS conversion value of 0.98 (95% CI: −1.1–3.1) in comparison to the atezolizumab plus bevacizumab combination therapy, with no statistically significant difference observed. The efficacy rankings, from highest to lowest, were atezolizumab plus bevacizumab, lenvatinib, sorafenib, sorafenib plus pravastatin, cabozantinib, pravastatin, and placebo.

### 3.3 Safety

**FIGURE 4:**
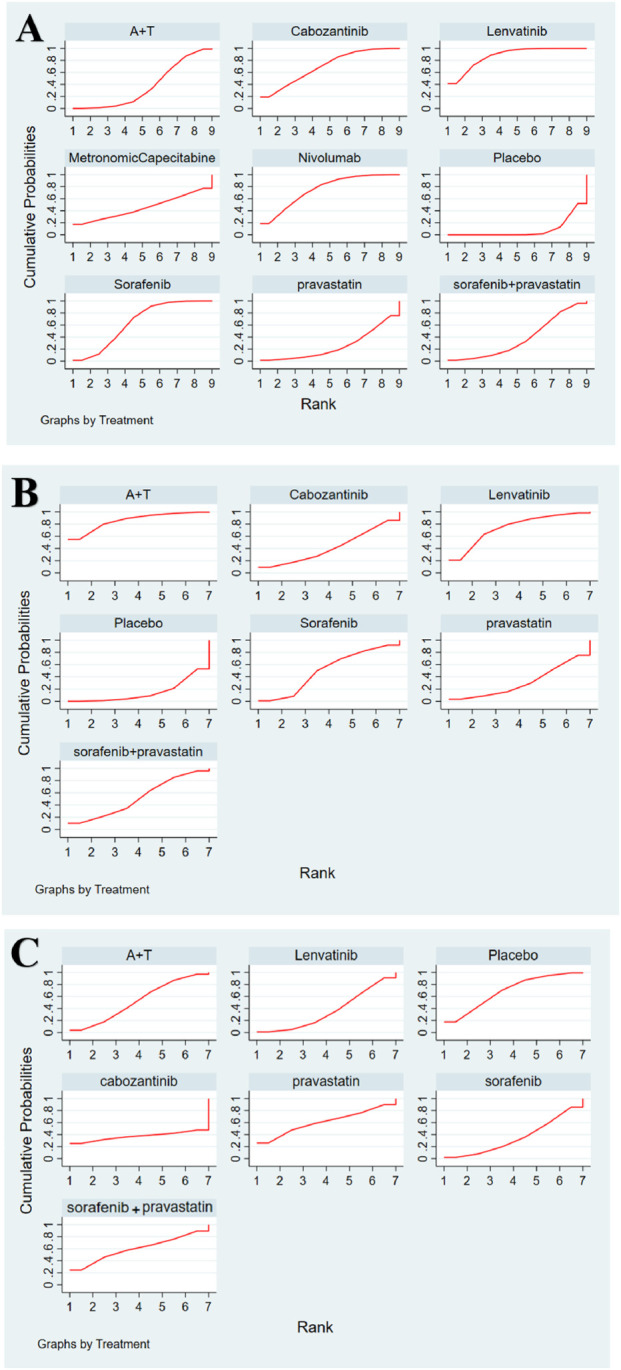
Cumulative graph of probability. **(A)** Overall survival. **(B)** Progression-free survival. **(C)** AEs.

Seven studies reported AEs across seven dose regimens, including 1,117 individuals, of whom 776 experienced AEs of varying severity, resulting in an overall incidence of 68.58%. The predominant grade 3–4 AEs were hypertension, proteinuria, hand-foot syndrome, and abnormal liver function. The cumulative probability plot (Figure 4C) of the safety analysis indicated that the combination of sorafenib and pravastatin exhibited the lowest incidence of AEs of any grade. The SUCRA value for sorafenib monotherapy was 59.9 (Figure 3C), while that for atezolizumab plus bevacizumab combination therapy was 52.8. The incidence of AEs for lenvatinib monotherapy was marginally lower than that for sorafenib monotherapy, with the most prevalent adverse reactions being loss of appetite, followed by fatigue, abnormal liver function, proteinuria, and hypertension.

## 4 Discussion

This NMA indirectly compared first-line systemic therapy for patients with advanced HCC and Child-Pugh class B hepatic function. Survival data for 2,536 individuals were gathered from 11 studies, and nine first-line therapy regimens were comprehensively evaluated regarding efficacy and safety in patients with HCC and hepatic function classified as Child-Pugh class B. For Child-Pugh class B patients, owing to their poorer liver function status, the incidence of AEs at all grades was high, and the therapeutic effects of various drug treatments were generally unsatisfactory, with minimal differences between treatment options. The OS and PFS periods were relatively short in these patients, with lenvatinib demonstrating the most favorable OS outcomes. The combination of atezolizumab plus bevacizumab proved most effective for prolonging PFS. The combination regimen of sorafenib and pravastatin exhibited reduced toxicity compared to atezolizumab plus bevacizumab combination therapy in terms of safety. While sorafenib combined with pravastatin was identified as the safest treatment regimen, this NMA unequivocally indicated that it was inferior in OS and PFS compared with atezolizumab plus bevacizumab combination therapy, as well as lenvatinib and nivolumab monotherapy regimens. Ultimately, in comparison with atezolizumab plus bevacizumab, lenvatinib monotherapy demonstrated a superior net health benefit regarding OS, albeit with a heightened risk of toxicity. Regarding PFS, the net health benefit favored lenvatinib.

Atezolizumab plus bevacizumab combination therapy is now regarded as the standard first-line treatment for advanced HCC, demonstrating substantial enhancements in OS, PFS, and quality of life compared with sorafenib monotherapy (Cheng et al., 2022). In the literature reviewed, individuals with advanced HCC classified as Child-Pugh B have only been subjected to direct comparisons between atezolizumab plus bevacizumab, lenvatinib, nivolumab, and placebo. The effects of atezolizumab plus bevacizumab on OS were not significantly different from those of treatments other than lenvatinib monotherapy, as determined by indirect comparisons using NMA. Furthermore, Chapin et al. (2023) demonstrated that nivolumab exhibited superior OS and tolerability compared to sorafenib in patients with HCC and Child-Pugh B cirrhosis, with a statistically significant difference in OS. Notably, nivolumab may provide greater benefits for patients with poorer performance status. Additionally, although the difference was not statistically significant, nivolumab showed a trend towards lower discontinuation rates compared to sorafenib due to toxicity or clinical deterioration. This may be attributed to the substantial overlap between the common adverse events associated with sorafenib (e.g., fatigue, anorexia, and nausea) and the clinical manifestations of HCC progression or hepatic decompensation, potentially leading to misclassification during the assessment. Such diagnostic challenges are particularly pronounced in patients with advanced HCC and Child-Pugh B cirrhosis. The findings of Fulgenzi et al. (2024) align with our results, indicating no significant differences in OS, PFS, or overall treatment duration between atezolizumab plus bevacizumab (A + T) and nivolumab in cohorts with well-balanced baseline characteristics. As a PD-1 inhibitor, nivolumab demonstrated favorable efficacy and safety profiles in this meta-analysis, suggesting that immune monotherapy retains certain advantages in Child-Pugh B patients. Although nivolumab showed a numerical trend towards improved OS, this difference warrants cautious interpretation. Given the limited number of studies evaluating nivolumab in this population, further high-quality research is needed to validate the efficacy and safety of immunotherapy in this special subgroup. Future prospective studies should rigorously investigate the comparative effectiveness of different immunotherapy regimens in the Child-Pugh B population, with strict control of baseline liver function, tumor burden, and etiological factors, to corroborate these findings.

Patients classified as Child-Pugh B exhibit markedly compromised liver function, leading to diminished drug metabolism and clearance, thereby increasing their vulnerability to adverse drug reactions. Our investigation revealed that lenvatinib demonstrated a superior equilibrium between efficacy and safety. In our study, lenvatinib was deemed most likely to rank highest in efficacy among all treatments, except for atezolizumab plus bevacizumab combination therapy, aligning with the findings of Rimini et al. (2023). In contrast, our PFS results demonstrated no statistically significant difference between the two regimens. It has been posited that PFS may be overestimated in instances of high treatment toxicity due to end-of-treatment information bias, typically arising from patients who prematurely withdraw from the trial due to serious AEs. Although the atezolizumab plus bevacizumab combination therapy demonstrated better tolerability in terms of AEs, the safety profile of lenvatinib, which was comparable to that of sorafenib, was acceptable. Mechanistically, as a multi-target tyrosine kinase inhibitor, lenvatinib exerts its antitumor effects through simultaneous inhibition of the vascular endothelial growth factor receptor, fibroblast growth factor receptor, and platelet-derived growth factor receptor pathways. Importantly, its efficacy remains independent of intact immune function, presenting a distinct advantage over PD-L1 inhibitors, whose therapeutic effects may be compromised in patients with pre-existing immune dysfunction such as T-cell exhaustion and lymphopenia. Furthermore, compared to bevacizumab, which carries significant risks of worsening ascites and gastrointestinal bleeding in patients with portal hypertension, lenvatinib demonstrates a more favorable safety profile. These pharmacological characteristics collectively explain its superior clinical performance and support its use as a preferred first-line treatment for HCC, particularly in Child-Pugh B patients whose hepatic functional reserve limits therapeutic options. These evidence-based findings have important implications for clinical decision-making in this challenging patient population.

Our safety data from the NMA indicated that the combination regimens (atezolizumab plus bevacizumab and sorafenib plus pravastatin) correlated with a minimal risk of AEs compared with monotherapies. The prevalent AEs of sorafenib were fatigue and diarrhea, which diminished the likelihood of hepatic function problems when administered alongside pravastatin. The favorable safety profile of sorafenib and pravastatin combination therapy was counterbalanced by its limited efficacy. The prevalent AEs of atezolizumab plus bevacizumab, such as liver function abnormalities and anorexia, remained safe compared to sorafenib. In patients where PFS benefit is prioritized and no obvious portal hypertension or ascites is observed, the atezolizumab plus bevacizumab should be given priority consideration. Nevertheless, routine monitoring for immune-related adverse events remains necessary, and once such events occur, immunosuppressive therapy must be promptly initiated. The predominant AEs of lenvatinib were anorexia, followed by fatigue, hepatic dysfunction, proteinuria, and hypertension. However, when lenvatinib is selected, close monitoring of proteinuria is essential, particularly in patients with pre-existing renal diseases at baseline. Additionally, monitoring of bilirubin levels and signs of hepatic encephalopathy is required, especially in patients with a history of ascites or hepatic encephalopathy at baseline. In the context of pharmacotherapy for patients with Child-Pugh class B liver function, priority should be given to the protection of liver function. During the treatment period, indicators such as bilirubin, albumin, and coagulation function must be closely monitored to prevent further deterioration of liver function.

### 4.1 Limitations

This study had some limitations. First, due to the innovative nature of this study, there are few studies of patients with HCC and Child-Pugh B liver function, and the proportion of patients included in the study and Child-Pugh ratings varied, which may have affected the accuracy and credibility of the results. Second, the lack of individual patient data prevented us from performing more in-depth subgroup analyses, such as the effect of different etiologies or tumor loads on treatment outcomes. Finally, uncertainty in model selection and parameter estimation due to the complexity of the NMA may have also impacted the results.

## 5 Conclusion

In summary, our NMA analysis utilizing individual patient data for first-line systemic therapy regimens in advanced patients with HCC and Child-Pugh B liver function showed that (i) atezolizumab plus bevacizumab combination therapy demonstrated the most favorable net benefit for PFS and reduced toxicity, thereby warranting its recommendation as a standard-of-care regimen. (ii) Compared to atezolizumab plus bevacizumab, lenvatinib monotherapy offers the greatest net improvement in OS and may be a suitable alternative when physicians and patients are prepared to tolerate a higher risk of AEs.

## Data Availability

The original contributions presented in the study are included in the article/Supplementary Material, further inquiries can be directed to the corresponding author.
