# Supplementary material for: A network meta-analysis of first-line treatment options for patients with Child-Pugh class B functional hepatocellular carcinoma: comparison of efficacy and safety
Source: Front Pharmacol. 2026 Jan 2;16:1705502. doi: 10.3389/fphar.2025.1705502 (PMC12807933; doi:10.3389/fphar.2025.1705502)
Supplement: Supplementary file 1 [file Supplementaryfile1.docx]

Supplementary Material

# Supplementary Figures and Tables

## Supplementary Figures


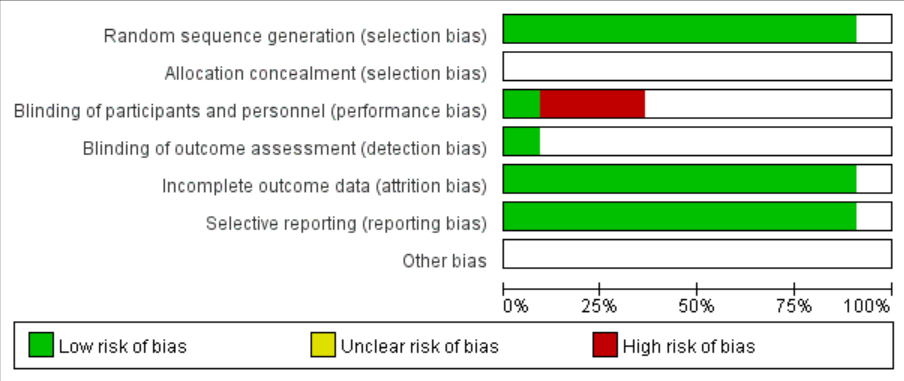


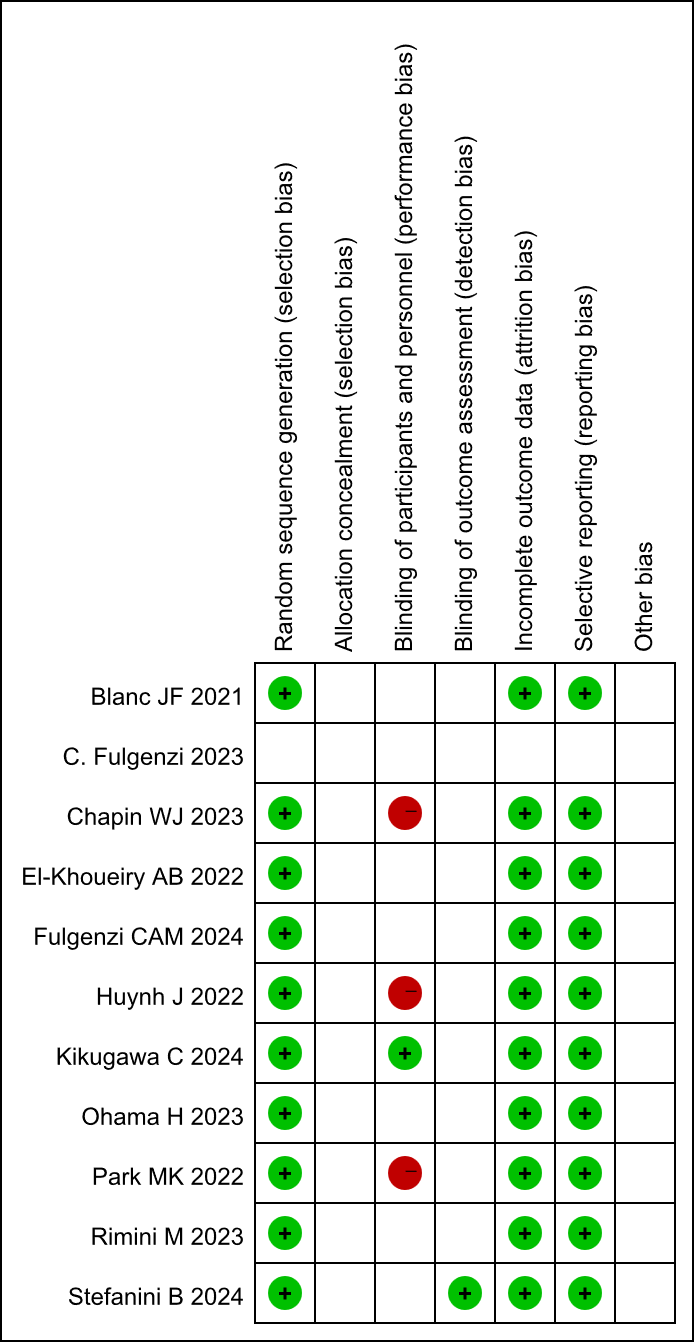


**Supplementary Figure 1.** Risk of bias graph for included studies. A. Risk of bias graph: review authors' judgements about each risk of bias item presented as percentages across all included studies. B. Risk of bias summary: review authors' judgements about each risk of bias item for each included study.

**Supplementary Figure 2.** League table. A. Overall survival. B. Progression-free survival.


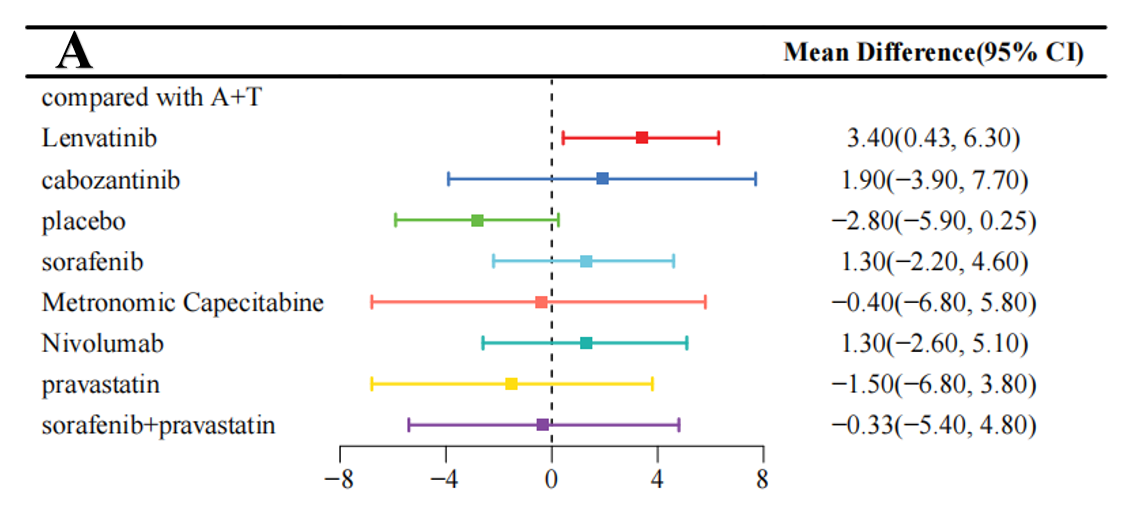


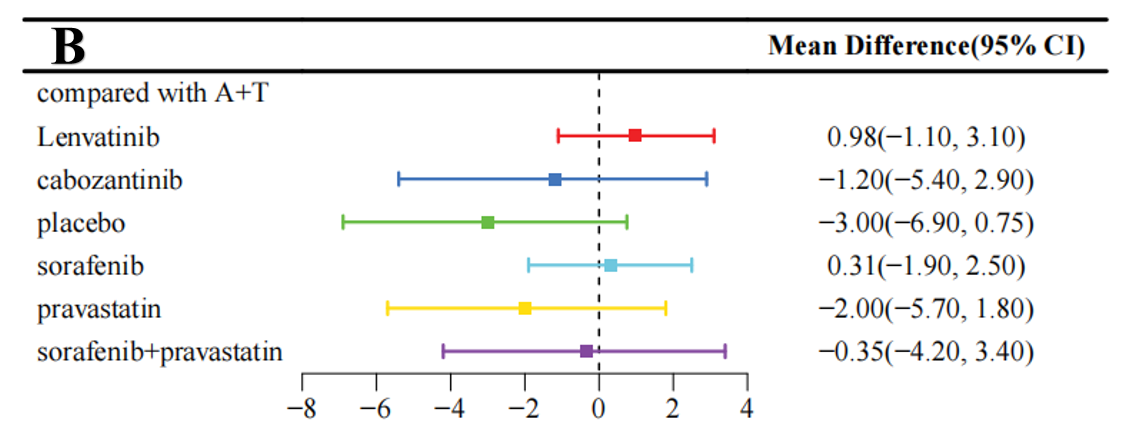


**Supplementary Figure 3.** Forest plot of network meta-analysis using random-effects model. A. Overall survival. B. Progression-free survival.
